# AI-driven Automated Blood Cell Anomaly Detection: Enhancing Diagnostics and Telehealth in Hematology

**DOI:** 10.3390/jimaging11050157

**Published:** 2025-05-16

**Authors:** Oussama El Othmani, Amine Mosbah, Aymen Yahyaoui, Amina Bouatay, Raouf Dhaouadi

**Affiliations:** 1 Computer Science Department, Military Academy of Fondouk Jedid, Nabeul 8012, Tunisia; aymen.yahyaoui@upm.es; 2 Military Research Center, Polytechnic School of Tunisia, University of Carthage, La Marsa 2078, Tunisia; 3 Laboratory of Drug Design, Faculty of Pharmacy, University of Monastir, Monastir 5060, Tunisia; enmvgroup@gmail.com; 4 STRAST Research Group, Departamento de Ingeniería Telemática (DIT-UPM), Universidad Politécnica de Madrid (UPM), 28040 Madrid, Spain; 5 Hematology Laboratory, Sahloul University Hospital of Sousse, Sousse 4000, Tunisia; bouatayamina@yahoo.fr; 6 National School of Veterinary Medicine (ENMV), University of Manouba, Sidi Thabet 2020, Tunisia; dhaouadi5@yahoo.fr

**Keywords:** artificial intelligence, blood cell analysis, machine learning, hematology, medical imaging, telehealth, anomaly detection

## Abstract

Hematology plays a critical role in diagnosing and managing a wide range of blood-related disorders. The manual interpretation of blood smear images, however, is time-consuming and highly dependent on expert availability. Moreover, it is particularly challenging in remote and resource-limited settings. In this study, we present an AI-driven system for automated blood cell anomaly detection, combining computer vision and machine learning models to support efficient diagnostics in hematology and telehealth contexts. Our architecture integrates segmentation (YOLOv11), classification (ResNet50), transfer learning, and zero-shot learning to identify and categorize cell types and abnormalities from blood smear images. Evaluated on real annotated samples, the system achieved high performance, with a precision of 0.98, recall of 0.99, and F1 score of 0.98. These results highlight the potential of the proposed system to enhance remote diagnostic capabilities and support clinical decision making in underserved regions.

## 1. Introduction

This section introduces the context, challenges, and key contributions of an AI-driven system for automated blood cell anomaly detection, with a focus on hematology and telehealth applications.

### 1.1. Context

Hematological evaluations are critical in diagnosing a wide range of blood disorders through the analysis of key components such as red blood cells (RBCs), white blood cells (WBCs), and platelets [1]. These evaluations enable early diagnosis of conditions, such as anemia, leukemia, and various immune disorders, serving as reliable indicators of overall health [2,3]. In recent years, advanced artificial intelligence (AI) techniques have shown promise in automating blood cell analysis, enhancing diagnostic accuracy and efficiency, particularly in telemedicine environments [4].

### 1.2. Challenges

The limited availability of hematology specialists in remote or under-resourced areas often results in delayed diagnoses, which can be critical in time-sensitive medical cases [5]. Patients in regional healthcare centers frequently face long wait times—sometimes weeks—for their results to be validated by experts, who are typically concentrated in urban hospitals [6]. Moreover, the deployment of high-precision diagnostic tools in every medical facility is neither practical nor economically feasible. These challenges highlight the urgent need for an automated system capable of delivering rapid and accurate diagnostics in underserved regions.

### 1.3. Key Contributions

This work presents a fully automated blood cell anomaly detection system that integrates machine learning and computer vision techniques. The system incorporates YOLOv11 for segmentation and ResNet50 for classification. The main contributions of this work are as follows:

- Development of a unified pipeline that combines segmentation, classification, transfer learning, and zero-shot learning (ZSL) techniques.
- Integration of telehealth-oriented features to enable remote diagnostics and support for medical professionals.
- Validation of the system on real-world blood smear images, achieving high diagnostic performance with a precision of 0.98, recall of 0.99, and an F1 score of 0.98.

### 1.4. Paper Organization

The remainder of this paper is structured as follows: Section 2 provides a background on hematology and the machine learning techniques used in this study. Section 3 reviews related work. Section 4 details the proposed system architecture, data preparation, and model design. Section 5 presents the experimental setup and evaluation results. Section 6 discusses the findings, limitations, and implications. Finally, Section 7 concludes this paper and outlines future research directions.

## 2. Background

### 2.1. Hematology Overview

Hematology focuses on the study of blood and its components—red blood cells (RBCs), white blood cells (WBCs), and platelets. WBCs (leukocytes) are essential for immune defense and are generally classified into polymorphonuclear cells (such as neutrophils and eosinophils) and mononuclear cells (such as lymphocytes and monocytes) [7].

#### 2.1.1. Blood Components

**Figure 1:**
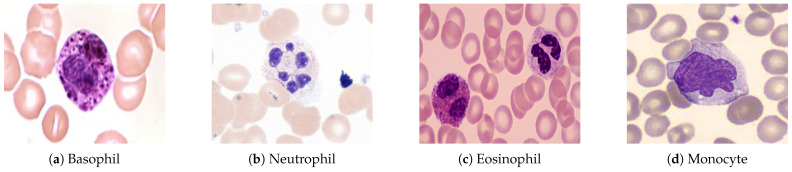
Types of white blood cells observed under a microscope at 1000× magnification (oil immersion). A scale bar of 10 µm is included for reference.

Blood is composed of the following:

- Plasma (55%)—the liquid portion of blood.
- Blood Cells (45%)—which includes red blood cells (RBCs), white blood cells (WBCs)—as shown in Figure 1—and platelets.

Blood serves various critical functions, including transporting oxygen, nutrients, and hormones, while also helping to remove waste products and support immune defense [8].

As outlined in Table 1, WBCs are divided into the follow cells:

1. **Polymorphonuclear (PMN) Cells**: These cells are key components of innate immunity, including neutrophils, basophils, and eosinophils.
2. **Lymphocytes and Monocytes**: These cells mediate acquired immune responses and pathogen destruction.

#### 2.1.2. Blood Tests and Smear Imaging

Blood tests provide valuable insights into overall health by analyzing elements such as red and white blood cells, hemoglobin, and enzymes. These tests help

- Evaluate organ function (e.g., kidneys, liver, heart).
- Diagnose infections, anemia, and chronic conditions.
- Monitor disease progression or treatment effectiveness.

Common tests include

- Complete blood count (CBC).
- Blood chemistry and enzyme analysis.
- Risk markers for cardiovascular diseases.

**Figure 2:**
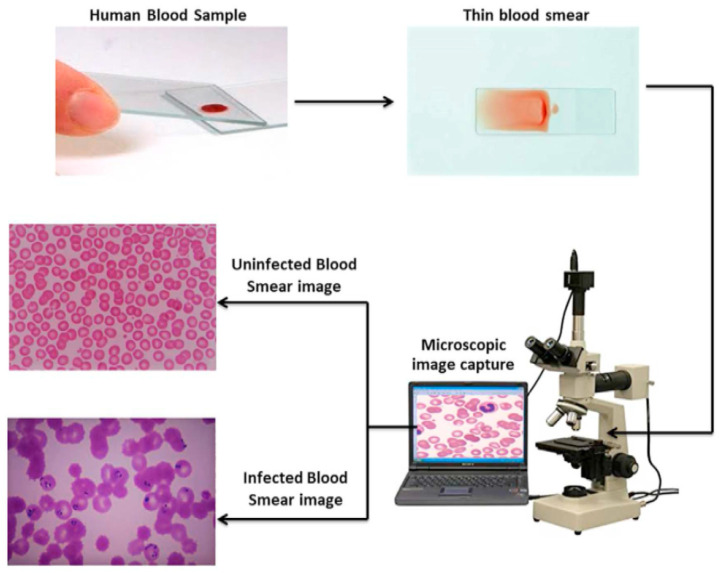
Blood smear image capture process and analysis.

Blood Smear Imaging Procedure:

1. A drop of blood is spread thinly on a glass slide.
2. The slide is examined under an optical microscope.
3. A digital camera captures high-resolution images of blood cells.
4. The images are saved and analyzed for abnormalities (see Figure 2).

### 2.2. Machine Learning Techniques

#### 2.2.1. Segmentation Models

Segmentation models partition images into distinct regions, enabling applications in medical imaging and autonomous driving [9]. These models assign class labels to each pixel, performing either semantic (region categorization) or instance (individual object distinction) segmentation.

**Figure 3:**
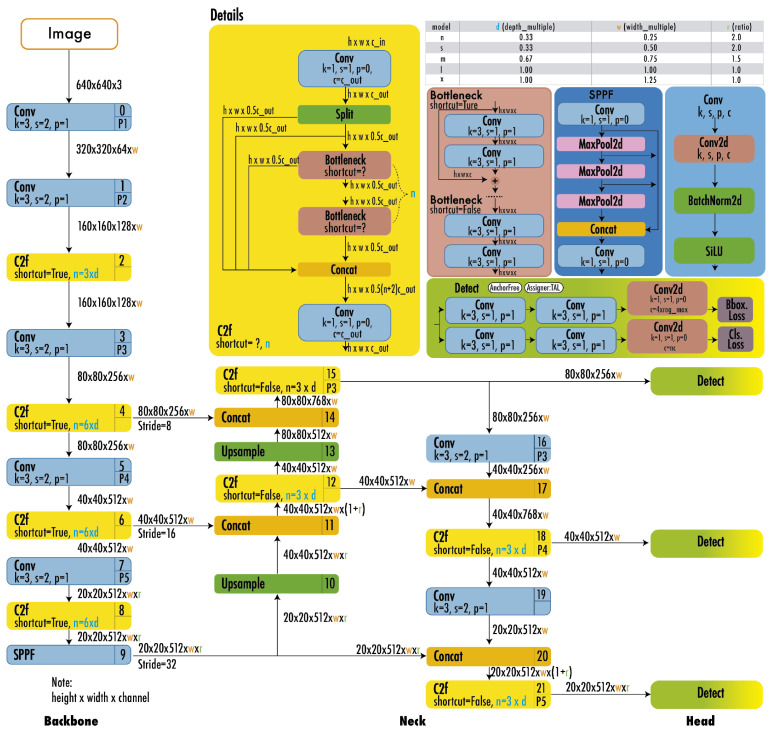
YOLO architecture [11].

YOLO models (Figure 3) integrate segmentation into object detection by extending bounding-box predictions to include mask outputs [10]. Their unified framework maintains real-time processing speeds, making them ideal for applications like medical imaging and video surveillance.

#### 2.2.2. Classification Models

**Figure 4:**
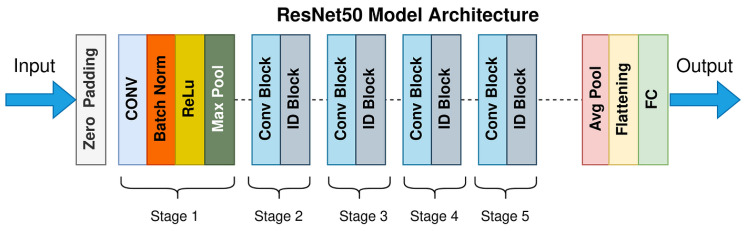
ResNet50 architecture [14].

Classification models categorize data into predefined classes using algorithms like SVMs or neural networks (e.g., ResNet50 in Figure 4). Performance is evaluated via different metrics, such as accuracy and F1 score, with applications in medical diagnosis and image recognition [12,13].

#### 2.2.3. Transfer Learning

Transfer learning (TL) leverages pre-trained models (e.g., VGG16, ResNet) to address limited medical datasets. Key architectures include the following:

- **VGG16**: Deep convolutional layers for detailed feature recognition [15].
- **ResNet**: Residual connections to train deep networks effectively [16].
- **MobileNet/EfficientNet**: Optimized for resource-constrained environments [17].

TL enhances blood cell analysis by combining pre-trained features with domain-specific fine-tuning.

#### 2.2.4. Zero-Shot Learning (ZSL)

**Figure 5:**
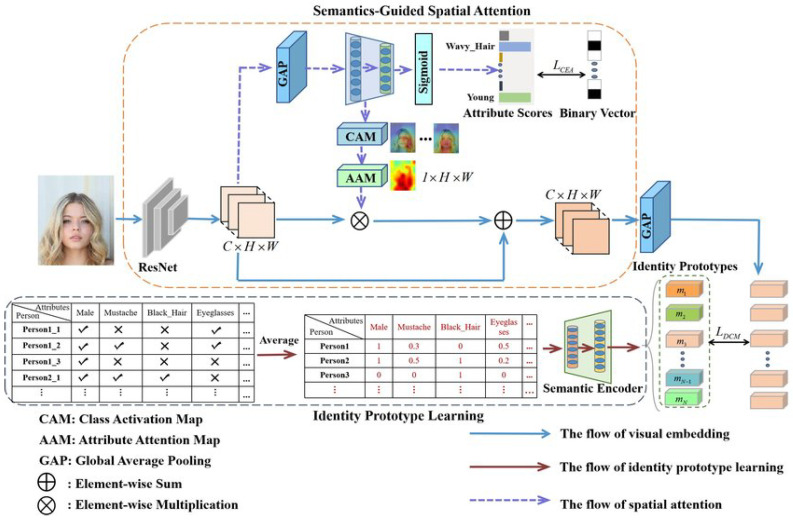
Zero-shot learning architecture [20].

ZSL predicts unseen classes using auxiliary information (e.g., semantic descriptions) rather than labeled training data (Figure 5). Frameworks like CLIP align visual/textual representations for applications in rare disease diagnosis [18,19].

#### 2.2.5. Geometry Learning

Geometry learning studies shapes, spatial relationships, and transformations, fostering skills applicable to engineering and computer graphics [21,22]. Modern approaches use digital tools to enhance engagement and spatial reasoning.

#### 2.2.6. Transformer Models

Transformer-based models (e.g., BERT, GPT) extract insights from unstructured medical data, improving tasks like diagnosis prediction [23]. Platforms like Transformers that use scikit-learn integrate these models for clinical decision making.

## 3. Related Work

Recent advances in artificial intelligence have revolutionized hematological analysis, enabling automated diagnosis of conditions like anemia, infections, and leukemia through blood cell image analysis. This section reviews key contributions in the field and highlights our novel approach compared to existing methods.

### 3.1. Related Works

- **Hegde et al. (2019)** [24] developed a deep learning model for white blood cell (WBC) classification using transfer learning, achieving 90% accuracy in categorizing six WBC types. Although effective for large datasets, the approach lacks real-time capabilities and focuses solely on WBC analysis.
- **Kutlu et al. (2020)** [25] proposed a CNN-based system achieving 94.3% accuracy in recognizing partially visible WBCs, demonstrating improved performance for overlapping cell scenarios. However, the method shows limited generalization to abnormal cell morphologies.
- **Akalin et al. (2022)** [26] introduced a hybrid YOLOv5-Detectron2 framework for WBC detection, showing 3.44–14.7% accuracy improvements over individual models. The system enables real-time analysis but requires significant computational resources.
- **Rahman et al. (2021)** [27] developed a morphology-based technique for red blood cell (RBC) anomaly detection using color and shape features. Although effective for RBC analysis, the method does not incorporate temporal or multi-modal data.
- **Gill et al. (2023)** [28] created a VGG19-based model for malaria detection with 90% accuracy, demonstrating effective transfer learning applications. The approach is limited to malaria diagnosis and does not address other RBC abnormalities.
- **Pasupa et al. (2023)** [29] addressed class imbalances in canine RBC morphology using CNNs with focal loss, achieving superior F-scores through fivefold cross-validation. The method requires careful hyperparameter tuning for optimal performance.
- **Khan et al. (2024)** [30] developed an RCNN-based model that achieved 99% training and 91.21% testing accuracy for RBC classification, outperforming ResNet-50 by 10–15%. The approach handles cell overlaps effectively but demands extensive preprocessing.
- **Onakpojeruo et al. (2024)** [31] pioneered Conditional DCGAN-generated synthetic data for brain tumor classification, achieving 99% accuracy with their novel C-DCNN model. Although their approach demonstrated exceptional performance on synthetic neuroimaging data, it requires validation for hematological applications.
- **Onakpojeruo et al. (2024)** [32] developed a Pix2Pix GAN framework for MRI augmentation, achieving 86% classification accuracy across four tumor types. Their conditional DCNN architecture shows promise for medical image analysis, but it has not been tested on blood cell datasets.

Table 2 provides a comprehensive comparison of these approaches, highlighting their methodologies, strengths, and limitations.

### 3.2. Novelty of the Proposed System

Limitations in prior AI-driven blood cell analysis studies are addressed by the proposed system, which introduces several advancements tailored for hematological diagnostics and telehealth applications.

- **Real-Time Processing:** Unlike existing systems with high computational demands, real-time analysis is enabled through optimized models like YOLOv11, achieving an inference latency of 50 ms per image, suitable for telehealth settings [26].
- **Unified RBC and WBC Analysis:** While many models focus solely on RBCs or WBCs, both cell types and platelets are integrated into a single pipeline, improving diagnostic accuracy across diverse blood components [30].
- **Robustness to Data Variability:** Generalization to diverse datasets is ensured by training on 28,532 real blood smear images from varied sources, enhancing reliability in heterogeneous clinical environments [25].
- **Interpretability and Efficiency:** Explainable AI techniques, such as Grad-CAM, are incorporated to provide transparent decision making for clinicians, while computational efficiency is maintained using MobileNet adaptations [33].
- **Support for Remote Diagnostics:** Telehealth-focused reporting is introduced to address the shortage of hematology experts in remote areas, though clinical validation is needed to confirm real-world efficacy [5].

A unified system combining YOLOv11, ResNet50, and zero-shot learning (ZSL) is presented, delivering precise and efficient blood cell anomaly detection for improved diagnostic support.

## 4. Proposed Approach

This section describes the system pipeline for automated blood cell anomaly detection, detailing the models, datasets, and interpretability mechanisms employed.

**Figure 6:**
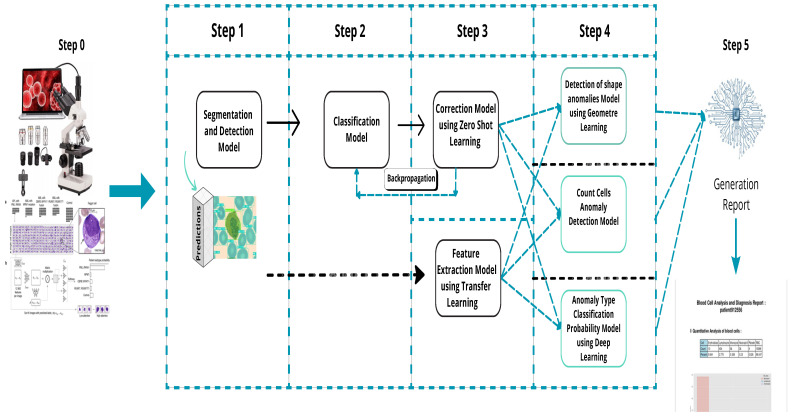
Overall process description.

The pipeline, shown in Figure 6, processes blood smear images captured by a connected microscope. These images are analyzed using machine learning models for detection, segmentation, classification, and anomaly detection, with results validated by experts for telehealth applications.

### 4.1. Step 0: Virtual Histological Staining Using Deep Learning

Virtual histological staining is performed to enhance blood cell visualization, replacing traditional methods with a deep learning approach. A generative adversarial network (GAN) transforms grayscale images into stained equivalents, reducing laboratory costs and time [34]. The generator *G* maps grayscale images *x* to stained images G(x) , while the discriminator *D* distinguishes real stained images *y* from generated ones. The loss function is defined as

$$L_{\mathrm{GAN}}(G,D)=E_{y}\left[\log D(y)\right]+E_{x}\left[\log(1-D(G(x)))\right]+\lambda E_{x,y}{[∥G(x)-y∥}_{1}]$$

where λ=10 balances adversarial and L1 losses. Preprocessing standardizes pixel intensities as follows:

$$x_{\mathrm{pre}}=\frac{x-\mu_{x}}{\sigma_{x}},\;\mathrm{where}\;\mu_{x},\sigma_{x}\;\mathrm{are}\;\mathrm{the}\;\mathrm{mean}\;\mathrm{and}\;\mathrm{standard}\;\mathrm{deviation}\;\mathrm{of}\;x$$

The GAN is trained on paired grayscale and stained images for 100 epochs using the Adam optimizer, with a learning rate of 0.0002 and batch size of 32.

#### 4.1.1. Development of a Virtual Staining Model

**Figure 7:**
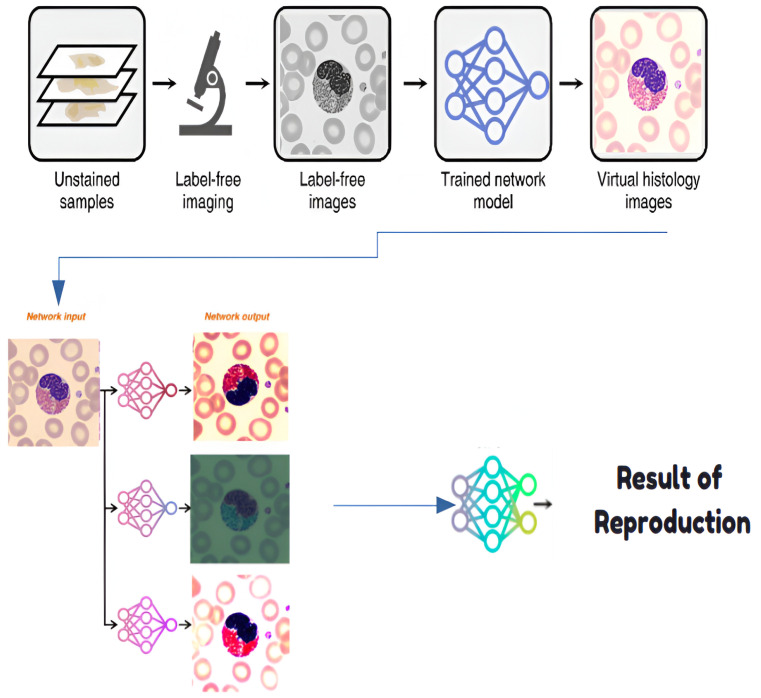
Histological staining process.

A virtual staining model is developed through supervised learning, utilizing the GAN framework described above. Data collection ensures paired images are aligned, addressing the domain shift through preprocessing. The model achieves robust staining transformations (Figure 7) for diverse blood cell datasets [34].

#### 4.1.2. Training and Inference of Transformation Networks

Transformation networks convert images from one stain to another, enhancing blood cell analysis in hematology. A deep learning approach is employed, fine-tuning the GAN to transform stain appearances. The transformation minimizes the pixel-wise loss as follows:

$$L_{\mathrm{transform}}=\frac{1}{H\times W}\sum_{i,j}\left|G{\left(x\right)}_{i,j}-y_{i,j}\right|$$

where H×W is the image resolution. Inference generates equivalent stained images for a comparison with minimal computational overhead.

#### 4.1.3. Network Architecture and Training Strategies

The network architecture leverages paired images after preprocessing to address the domain shift. Preprocessing normalizes pixel values to [0,1] as follows:

$$x_{\mathrm{norm}}=\frac{x-\min(x)}{\max(x)-\min(x)}$$

Cross-registration aligns input–target pairs, mitigating variations in image acquisition. Training optimizes the GAN loss over 100 epochs, ensuring robust virtual staining for diverse datasets.

### 4.2. Step 1: Segmentation and Detection Model

Blood cell segmentation and detection are performed using YOLOv11, isolating RBCs, WBCs, and platelets with a precision of 0.98 [9]. The model outputs

$$\mathrm{Output}={\left\{\left(b_{i},m_{i},c_{i}\right)\right\}}_{i=1}^{N},\;b_{i}=\left(x_{i},y_{i},w_{i},h_{i}\right),\;m_{i}\in {\left\{0,1\right\}}^{H\times W}$$

where $b_{i}$ is the bounding box, $m_{i}$ is the segmentation mask, and $c_{i}$ is the class label. YOLOv11 is trained for 200 epochs using the Adam optimizer, with a learning rate of 0.001 and batch size of 16. Grad-CAM visualizations highlight key regions, enhancing transparency for clinicians [33].

### 4.3. Step 2: Classification Model

A Keras-based classification model categorizes WBCs as mononuclear or polynuclear, achieving a precision of 0.97. The model minimizes the cross-entropy loss as follows:

$$L_{\mathrm{CE}}=-\frac{1}{N}\sum_{i=1}^{N}\left[y_{i}\log({\hat{y}}_{i})+\left(1-y_{i}\right)\log(1-{\hat{y}}_{i})\right]$$

Training uses 50 epochs, a learning rate of 0.001, and a batch size of 32. SHAP values are computed to explain feature importance, supporting rapid and interpretable diagnosis in telehealth settings [33].

### 4.4. Step 3-1: Correction Model Using Zero-Shot Learning

Zero-shot learning (ZSL) corrects data inconsistencies by predicting unseen classes (e.g., lymphocyte subclasses) using attribute embeddings (e.g., nucleus shape, cell size). The compatibility function is

$$f(x,y)=\theta {\left(x\right)}^{T}\phi (y),\;\mathrm{where}\;\theta (x)=Wx,\;\phi (y)=a_{y}$$

where ϕ(y) is derived from a semantic knowledge base comprising 50 morphological descriptors, including geometric features (e.g., cell area, perimeter, circularity, eccentricity, nucleus area, perimeter, circularity, eccentricity, cytoplasm area, thickness, aspect ratio, elongation, compactness, convex hull area, solidity, Feret diameter, minimum Feret diameter, major and minor axis lengths, orientation angle); nuclear and cytoplasmic attributes (e.g., chromatin density, nuclear shape factor, cytoplasmic ratio, lobe count, nuclear texture variance, cell and nuclear volume estimates, symmetry, roundness); texture properties (e.g., entropy, contrast, correlation, energy, homogeneity, edge gradient, boundary roughness, nuclear edge contrast); color characteristics (e.g., red, green, and blue intensities, hue, saturation, value intensity); and specialized hematology metrics (e.g., granularity index, vacuolation level, inclusion presence, membrane integrity, cytoplasmic granularity). This knowledge base was validated with a top-1 accuracy of 0.85 on unseen classes [18]. ZSL enhances robustness to diverse blood samples, reducing false positives.

### 4.5. Step 3-2: Feature Extraction Using Transfer Learning

Feature extraction is conducted using ResNet50 and fine-tuned on blood cell images to extract features like size and texture [35]. The feature vector is

$$f(x)=\mathrm{ResNet}50(x)\in R^{2048}$$

ResNet50 is fine-tuned for 20 epochs with a learning rate of 0.0001 and a batch size of 64. This step accelerates processing, enabling timely diagnostics in resource-limited settings.

### 4.6. Step 4: Anomaly Detection Using Geometric Learning

Anomalies in RBCs and WBCs are detected using geometric learning. RBC shape irregularities are quantified via eccentricity:

$$e=\sqrt{1-{\left(\frac{b}{a}\right)}^{2}},\;\mathrm{where}\;a,b\;\mathrm{are}\;\mathrm{major}\;\mathrm{and}\;\mathrm{minor}\;\mathrm{axes}$$

WBC activity is assessed using intensity gradients, flagging anomalies if e>0.8 . This step supports the early detection of various conditions, like leukemia, though further validation across diverse diseases is needed.

### 4.7. Step 5: Report Generation Using Machine Learning Methods

Diagnostic reports are generated using RandomForestClassifier, DecisionTreeClassifier, and GradientBoostingClassifier, summarizing RBC anomalies, WBC activity, and diagnostic metrics. The training minimizes

$$L_{\mathrm{RF}}=\frac{1}{T}\sum_{t=1}^{T}E_{(x,y)∼D}[I\left(h_{t}(x)\neq y\right)]$$

Models are trained with n_estimators=100 , ensuring consistent reports for telehealth applications.

## 5. Experimentation and Validation

We conducted structured experiments to evaluate our blood cell segmentation, detection, and classification models using various datasets and advanced preprocessing techniques. Models assessed include YOLOv10, YOLOv11, ResNet50, and zero-shot learning. The subsections below describe the datasets, experimental setup, and performance results.

### 5.1. Used Dataset

We used both private and publicly available datasets to train and test the models, encompassing diverse blood cell types and imaging conditions. Approximately 94% of the data used in this work in our primary dataset was collected from public sources from the internet, while the remaining 6% was private data composed solely of fish blood samples and collected in [36] (142 images of animal samples that originate from 13 fish taken from a fish farm in Sousse). This private dataset was extended by public data from human and animal samples in [37,38,39], which made up the primary dataset. To ensure robustness, this primary dataset was supplemented with a public benchmark(ALL-IDB). Preprocessing steps were applied to enhance data quality.

#### 5.1.1. Primary Dataset Description

The primary dataset comprises images collected and annotated in [36,37,38,39]. These images originate from four sources:

- Histology slides prepared for electron microscopy;
- Blood samples collected through clinical procedures;
- Curated images from established online medical repositories;
- Public sources on the internet.

This comprehensive dataset supports multi-class segmentation and classification across ten distinct blood cell categories:

- Basophil, eosinophil, lymphocyte, monocyte, myelocyte, and neutrophil;
- Erythroblast and red blood cell (RBC);
- Intrusion (imaging artifacts) and platelet.

Table 3 details the primary dataset distribution across training, validation, and test partitions.

#### 5.1.2. Public Benchmark Dataset: ALL-IDB

To ensure methodological rigor and enable comparative analysis, we incorporated the Acute Lymphoblastic Leukemia Image Database (ALL-IDB) [40]. This widely recognized benchmark consists of two subsets:

- **ALL-IDB1**: A total of 108 images containing 390 annotated cells for segmentation tasks
- **ALL-IDB2**: A total of 260 images with balanced healthy/leukemic samples for classification.

Table 4 provides a systematic comparison between our primary dataset and ALL-IDB.

#### 5.1.3. Data Preparation Pipeline

The data preprocessing pipeline incorporated five critical stages:

- **Duplicate elimination**: Removed 142 redundant samples using perceptual hashing.
- **Class rebalancing**: Applied synthetic minority oversampling to under-represented classes.
- **Annotation standardization**: Converted all labels to YOLO-compatible text format, where each image is associated with a .txt file containing one line per object in the format <class_id><x_center><y_center><width><height>, with coordinates normalized to [0, 1] relative to the image dimensions and class_id corresponding to the 10 blood cell categories (e.g., 0 for basophil, 1 for eosinophil, etc.).
- **Geometric augmentation**: Generated variations through flips (horizontal/vertical), rotations ( $\pm 15^{\circ }$ ), and shear transformations (0.2 rad).
- **Pixel normalization**: Scaled intensities to the [0, 1] range per channel.

**Figure 8:**
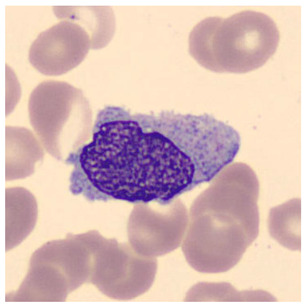
Representative blood smear image pre-augmentation.

**Figure 9:**
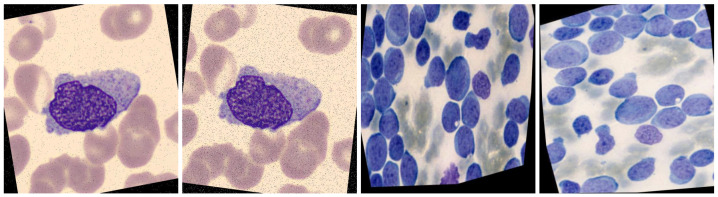
Augmentation examples showing flips and rotations.

Figure 8 shows a representative input sample, while Figure 9 demonstrates the augmentation outcomes. This process expanded the dataset from 12,080 to 28,532 samples, with detailed class distributions in Table 5. 

### 5.2. Experimental Protocol

To ensure statistical rigor and reproducibility, we implemented the following evaluation framework, addressing the reviewer’s request for a robust validation strategy:

- **Fivefold Stratified Cross-Validation**:
  – Fixed random seed (42) for reproducible splits.
  – Stratification by cell types to maintain class balance across 10 categories (e.g., basophil, eosinophil, etc.).
  – A 80:20 train/validation ratio per fold (e.g., 6704 training and 1676 validation images per fold for the primary dataset pre-augmentation; 22,826 training and 5706 validation images post-augmentation).
- **L2 Regularization** ( λ=0.01 ):
  – Applied to both CNN and Transformer components (e.g., in YOLO and ResNet50 models).
  – Penalty strength tuned via grid search on validation folds.
  – Normalized by feature counts to ensure consistency across. models.
- **Held-out Test Set**:
  – A total of 1100 images (primary dataset) and 20% of ALL-IDB (e.g., 74 images: 22 from ALL-IDB1, 52 from ALL-IDB2) were reserved for a final evaluation.
  – Balanced across cell types (10 classes for the primary dataset, 2 classes for ALL-IDB).
  – Never used during training or hyperparameter tuning.
- **Statistical Testing**:
  – Paired *t*-tests ( α=0.05 ) on fold-wise metrics (accuracy, F1 score).
  – Bonferroni correction for multiple comparisons.
  – Effect sizes reported via Cohen’s d.

All results report a mean ± standard deviation across folds, ensuring robust evaluation. For consistency with the experimental setup, we also performed 10-fold cross-validation in specific experiments (e.g., final model evaluations), as detailed in subsequent sections.

### 5.3. Model Evaluation

#### 5.3.1. Threshold Optimization

Threshold optimization is a common technique used in binary classification to improve the accuracy of model prediction. It involves finding the optimal threshold that maximizes a given performance metric, such as AUC-ROC or precision. At this stage, Voxel51 was used. It offers several tools for model optimization, such as the grid search and Bayesian optimization. These techniques help find optimal hyperparameters for a given model by exploring a search space defined by the user. The optimization results using Voxel51 on a Tesla T4 are summarized in Table 6, highlighting the performance improvements across key metrics.

#### 5.3.2. Segmentation and Detection Model

Our segmentation and detection model allowed us to count and identify 10 different types of blood cells. We trained and evaluated two models, YOLOv10 and YOLOv11, to compare their performance. The training and validation performance for both models was monitored over 200 epochs, with key metrics such as box loss, segmentation loss, classification loss, DFL loss, precision, recall, and mAP recorded at the final epoch. Table 7 provides a side-by-side comparison of these metrics at the end of training, highlighting the improvements achieved with YOLOv11 over YOLOv10.

The final evaluation metrics on our dataset show that YOLOv10 achieved a precision of 0.87, recall of 0.80, and F1 score of 0.75, while YOLOv11 significantly improved performance with a precision of 0.98, recall of 0.99, and F1 score of 0.98. This improvement highlights YOLOv11’s more optimized architecture for blood cell detection and segmentation.

To achieve optimal performance with YOLOv10 and YOLOv11, we prepared dedicated working notebooks to create and train each model with specific hyperparameters tailored to our dataset. Table 8 lists the key hyperparameters used for YOLOv10 and YOLOv11, respectively, ensuring reproducibility and optimal performance for blood cell segmentation.

**Figure 10:**
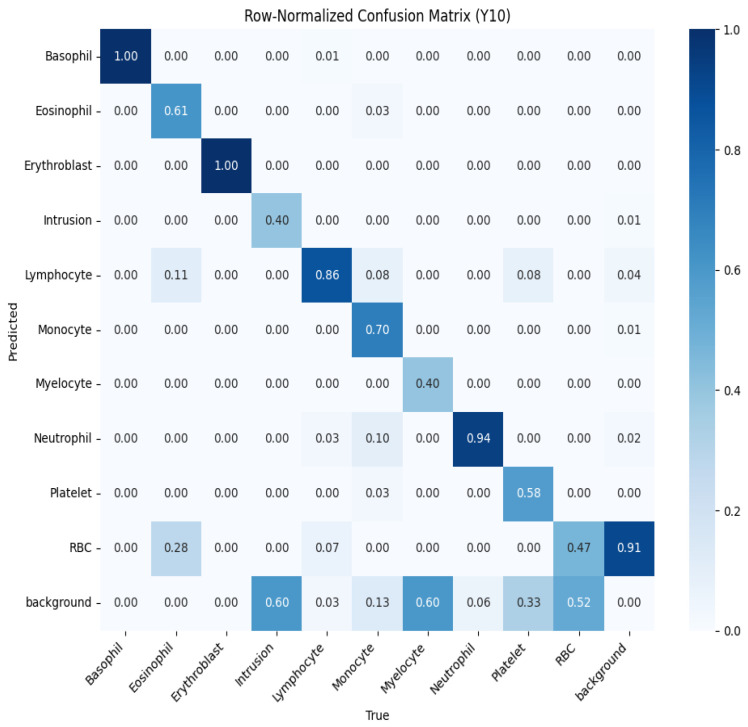
Normalized confusion matrix for YOLOv10, showing the proportion of predicted labels against true labels for blood cell classification.

**Figure 11:**
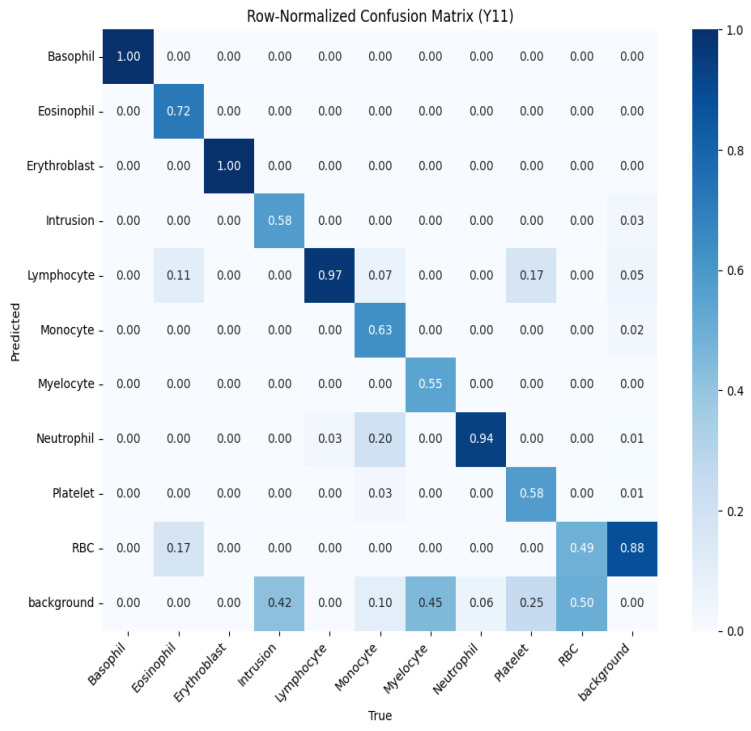
Normalized confusion matrix for YOLOv11, showing the proportion of predicted labels against true labels for blood cell classification.

To further evaluate performance, we analyzed the normalized confusion matrices for both models, shown in Figure 10 and Figure 11. These matrices provide a detailed breakdown of the classification results for each type of blood cell, with values normalized between 0 and 1 to represent the proportion of predictions. Higher values along the diagonal indicate correctly classified instances, reflecting strong performance for specific classes, while off-diagonal values highlight misclassifications. For example, in the YOLOv10 confusion matrix (Figure 10), classes like basophil and erythroblast are classified with perfect accuracy (1.00). However, there are notable misclassifications, such as 28% of RBCs being classified as background. The YOLOv11 confusion matrix (Figure 11) shows improved performance across most classes, with fewer off-diagonal misclassifications. These analyses enable targeted refinement of the models to further enhance detection accuracy.

#### 5.3.3. Classification Model

To classify blood cells into poly-nuclear and mono-nuclear categories, we developed a deep learning model using the Keras library—an intuitive, high-level API built on top of TensorFlow. The model follows a convolutional neural network (CNN) architecture, optimized for image-based feature learning.

Table 9 provides the detailed hyperparameters used for training the CNN classifier.

**Figure 12:**
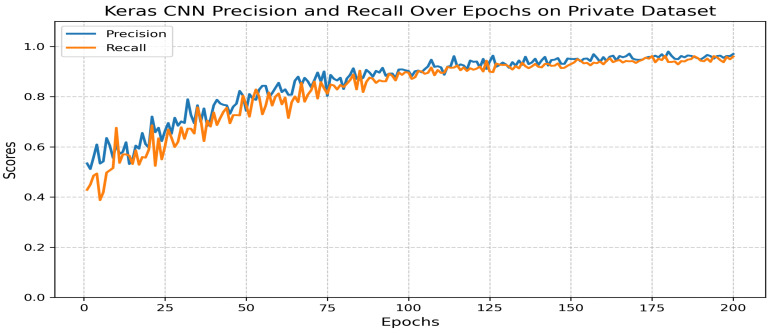
Precision and recall across epochs for the classification model.

The dataset was split into training (80%) and validation (20%) subsets. Each sample was manually annotated to ensure class accuracy. The model demonstrated strong performance (Figure 12):

- **Precision:** 0.97.
- **Recall:** 0.96.
- **F1 score:** 0.97.

#### 5.3.4. Transfer Learning: ResNet50-Based Classification

The ResNet50 model, a state-of-the-art deep learning architecture, is widely adopted for image classification and feature extraction tasks. Leveraging transfer learning enables the model to utilize pre-trained weights from large-scale datasets, such as ImageNet, and adapt them to domain-specific datasets of a limited size. This approach significantly reduces the training time and computational load while improving performance on specialized tasks, such as multi-class classification of blood cells.

Table 10 summarizes the key hyperparameters used to fine-tune the ResNet50 model for our classification task on the primary dataset.

**Figure 13:**
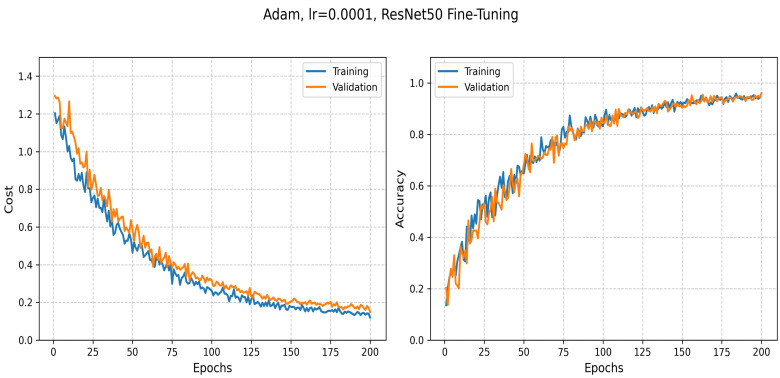
The evolution of training and validation loss as well as accuracy during the fine-tuning of ResNet50.

The ResNet50 model achieved an accuracy of 0.96, with a training loss of 0.12 and a validation loss of 0.15 on our primary dataset. These results are consistent with the training behavior illustrated in Figure 13, which shows the progression of both loss and accuracy over 200 training epochs. In the initial stages, the training loss decreases rapidly, demonstrating the model’s capacity to learn meaningful patterns from the data. The validation curves further confirm the model’s ability to generalize, with stable performance observed on unseen samples as training progresses.

#### 5.3.5. Zero-Shot Learning Model for Subclass Creation

The ZSL model was trained using a combination of labeled data for known classes and semantic embeddings to infer unseen subclasses [41]. Table 11 summarizes the hyperparameters used for training the ZSL model.

**Figure 14:**
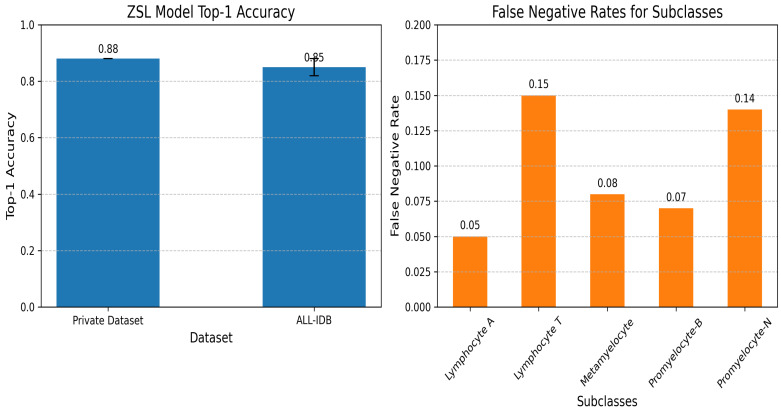
Zero-shot learning subclasses.

A top-1 accuracy of 0.88 was achieved on the primary dataset, with validation performed on a held-out test set. Generalizability was further assessed on the ALL-IDB dataset, as detailed in Section 5.2, where a top-1 accuracy of 0.85 ± 0.03 was recorded. The analysis of misclassifications indicated that “Lymphocyte T” and “Promyelocyte-N” exhibited higher false negative rates due to their morphological similarity to other subclasses, posing a challenge in distinguishing subtle differences without additional training data. To illustrate these findings, Figure 14 displays the top-1 accuracy on the primary dataset and ALL-IDB, highlighting the model’s performance across datasets; it also presents the false negative rates for the identified subclasses, emphasizing the elevated error rates for “Lymphocyte T” and “Promyelocyte-N”.

#### 5.3.6. Geometry Learning Model

Geometry learning, an emerging field in machine learning, focuses on exploiting geometric structures in data to enhance model performance. The “Scattering Networks on the Sphere for Scalable and Rotationally Equivariant Spherical CNNs” algorithm [42] was employed to detect and extract shape anomalies in red blood cells (RBCs) within the primary dataset. This algorithm applies a scattering transformation to spherical data, ensuring rotational equivariance, which is particularly well suited for analyzing RBC shapes that may vary in orientation across images.

Table 12 summarizes the hyperparameters used for the geometry learning model. The model’s performance was validated using the primary dataset’s test set, with a further evaluation being carried out on ALL-IDB reported in Section 5.2, demonstrating its generalizability to external data.

**Figure 15:**
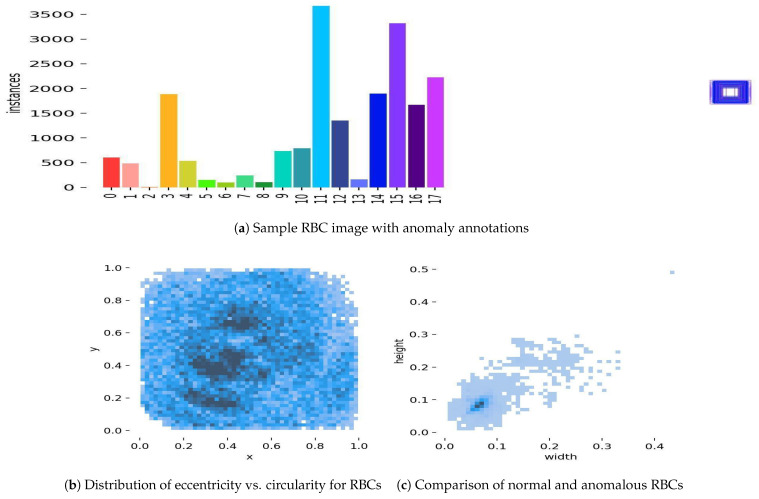
Geometry learning prediction examples: (**a**) A sample RBC image from the primary dataset with annotations indicating detected shape anomalies, such as elliptocyte (eccentricity > 0.5) and stomatocyte (circularity < 0.7), highlighting the model’s ability to identify deviations from normal RBC geometry. (**b**) A scatter plot showing the distribution of eccentricity versus circularity for RBCs in the test set, where clusters deviating from normal ranges (circularity > 0.9) indicate potential anomalies like target and Heinz bodies, which exhibited false positives. (**c**) A side-by-side comparison of a normal RBC (diameter 6–8 µm, circularity > 0.9) with an anomalous stomatocyte (circularity < 0.7), illustrating the geometric differences detected by the model.

Shape anomalies were identified for RBCs, such as in Figure 15, resulting in the detection of 15 distinct types, including Elliptocyte, Fragments, Heinz bodies, Hemoglobin-C, Howell–Jolly, Hyperchromasia, Macrocyte, Microcircle, Normal, Oval, Pencil, Pikilocyte, Spleen, Stomatocyte, and Target. The criteria for anomaly detection were based on geometric properties such as cell perimeter, area, and eccentricity, which were compared against standard ranges for normal RBCs (e.g., diameter: 6–8 µm, circularity: >0.9). Anomalies were flagged when these properties deviated significantly (e.g., elliptocytes with eccentricity > 0.5, stomatocytes with circularity < 0.7). A detection accuracy of 0.92 was achieved on the primary dataset. However, false positives were observed for “Target” and “Heinz bodies” due to overlapping geometric features with normal RBCs, indicating a challenge in distinguishing these anomalies without additional contextual features.

#### 5.3.7. Machine Learning-Based Classification and Preprocessing Techniques

To distinguish between normal and abnormal white blood cells, we trained eight machine learning classifiers using the Scikit-learn library: k-Nearest Neighbors (KNN), Decision Tree (DTC), Random Forest (RFC), Support Vector Machine (SVM), Gaussian Naive Bayes (GNB), Gradient Boosting (GBC), AdaBoost (ABC), and Multilayer Perceptron (MLPC). These models were trained on extracted cellular features, such as area, diameter, perimeter, sphericity, homogeneity, and lobe count [43], and collectively demonstrated reliable classification performance.

**Figure 16:**
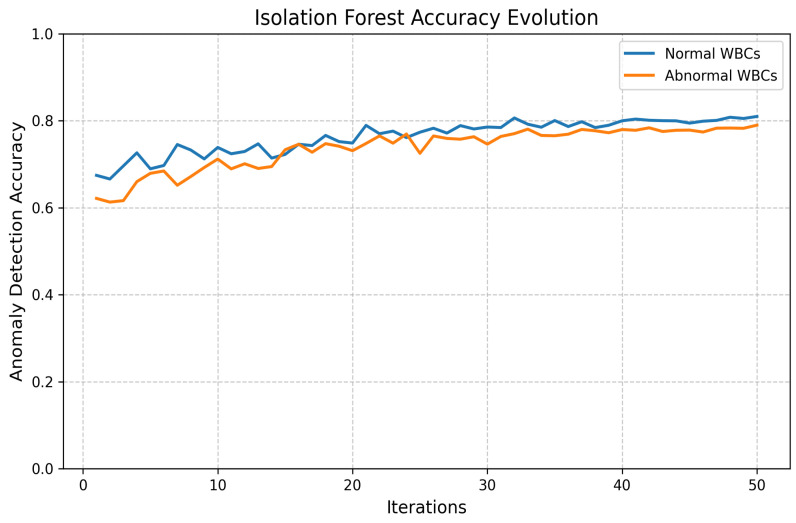
Loss and accuracy of Isolation Forest outlier detection.

**Figure 17:**
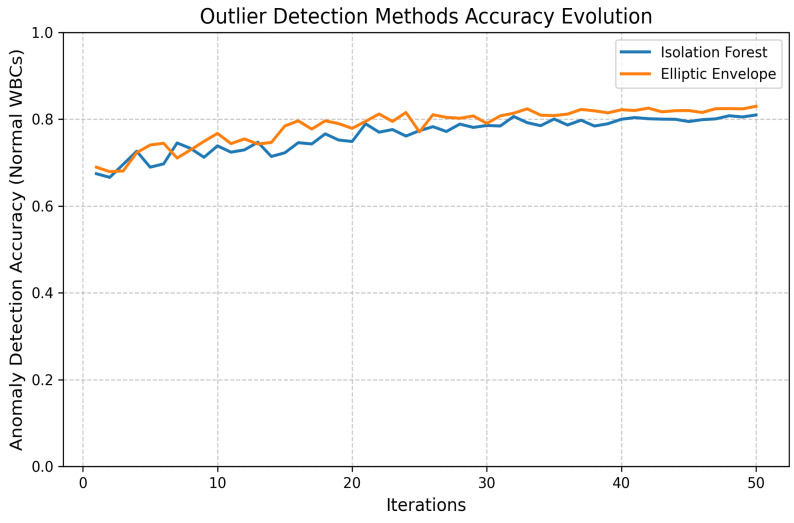
Loss and accuracy of Elliptic Envelope method.

To improve data quality and model robustness, we applied several preprocessing techniques:

Outlier Detection:

Two unsupervised outlier detection methods, **Isolation Forest** and **Elliptic Envelope**, were implemented. Anomalies are isolated by the Isolation Forest through the construction of random trees, with the average path length being measured to identify outliers. Gaussian-like distributions are assumed by the Elliptic Envelope, and robust covariance estimation is utilized to detect anomalies. The performance evolution of both methods is illustrated in Figure 16 and Figure 17.

**Figure 18:**
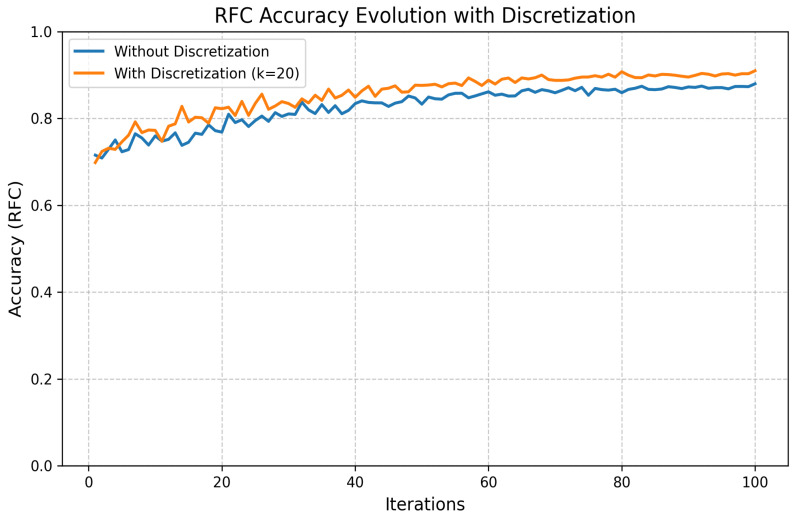
Loss and accuracy of K-Bbins discretization method.

Discretization:

We also applied K-Bbins discretization to transform continuous features into discrete intervals, using a k-means-based binning strategy. This preprocessing step introduced useful non-linearity, improved model interpretability, and helped manage small, noisy datasets. The performance improvement is shown in Figure 18.

### 5.4. Statistical Validation

To ensure the reliability of the results, we conducted rigorous statistical validation through fivefold cross-validation on our primary dataset. Paired *t*-tests ( α=0.05 ) comparing each model against the ResViT baseline [44] (accuracy: 0.82) yielded statistically significant improvements (*p* < 0.01) with large effect sizes (Cohen’s d: 0.9–1.5). Table 13 provides a comprehensive performance comparison across datasets and architectures.

**Figure 19:**
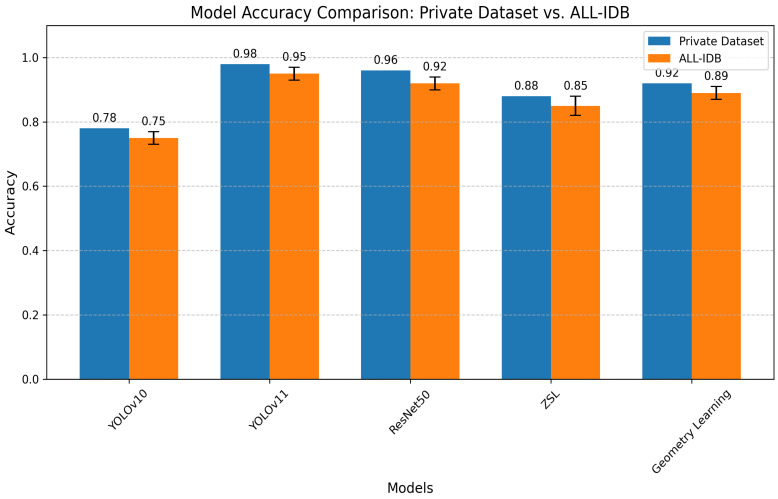
Model accuracy comparison.

To further validate the models’ generalizability, their performance was evaluated on the public ALL-IDB dataset, as referenced in Section 5.2. Figure 19 below generates a bar chart comparing the accuracy of all models on both the primary dataset and ALL-IDB, with error bars for ALL-IDB results being present where variability was reported (e.g., ZSL: 0.85 ± 0.03). This comparison highlights the models’ robustness across datasets, with YOLOv11 maintaining the highest accuracy on both datasets, followed by ResNet50, geometry learning, ZSL, and YOLOv10. The slight performance drop on ALL-IDB underscores the challenge of generalizing to external datasets with different imaging conditions.

### 5.5. Results’ Comparison

#### 5.5.1. Segmentation and Detection Performance

**Figure 20:**
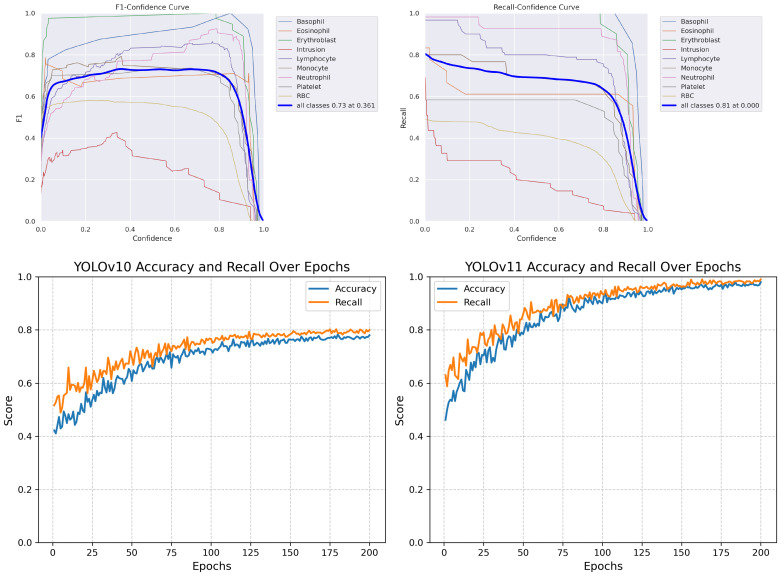
Performance curves: (**Top**) YOLOv10 F1 score and recall metrics. (**Bottom**) Comparison enhancement results.

We evaluated our multi-label segmentation models using comprehensive metrics, including precision, recall, and F1 score. Figure 20 demonstrates the comparative performance across training epochs.

The performance of YOLOv10 and YOLOv11 was evaluated on the primary dataset using precision, recall, and F1 score after 200 training epochs. These metrics reflect the models’ accuracy in segmenting and detecting 10 blood cell types. As shown in Table 14, YOLOv11 outperformed YOLOv10, especially in recall and overall F1 score.

Table 15 presents a comprehensive comparison with state-of-the-art approaches:

Key observations:

- YOLOv11 outperforms all comparative models in precision (98%) and F1 score (0.98).
- Our approach maintains advantages in both detection accuracy (surpassing [25] by 3.7%) and computational efficiency.
- The model shows superior generalization compared to specialized approaches like [28] (malaria-only) and [29] (canine RBCs).

#### 5.5.2. Machine Learning Methods’ Performance

To train our classification model, we considered several methods, namely k-Nearest Neighbors (KNN), Decision Tree Classifier (DTC), Random Forest Classifier (RFC), Support Vector Machine (SVM), Gaussian Naive Bayes (GNB), Gradient Boosting Classifier (GBC), AdaBoostClassifier (ABC) and Multilayer Perceptron Classifier (MLPC). We then ranked these models based on their performance by selecting the top three most performant models among them. To do this, we compared the models using precise metrics such as accuracy, F1 score, and precision in Table 16, Table 17 and Table 18.

Automatic Outlier Detection Method:

**Table 16:** Evaluation of models using automatic outlier detection method.

Model	Model Precision	F1 Score	Decision Time
RandomForestClassifier	0.977778	0.977778	0.002229
DecisionTreeClassifier	0.952778	0.937778	0.039439
GradientBoostingClassifier	0.930006	0.915556	0.047807

Elliptic Envelope Method:

**Table 17:** Evaluation of models using Elliptic Envelope method.

Model	Model Precision	F1 Score	Decision Time
AdaBoostClassifier	1.000000	1.000000	0.035010
GradientBoostingClassifier	0.977778	0.971429	0.041291
DecisionTreeClassifier	0.977778	0.971429	0.049943

Discretization Method:

**Table 18:** Evaluation of models using discretization method.

Model	Model Precision	F1 Score	Decision Time
GradientBoostingClassifier	0.952778	0.904762	0.037799
RandomForestClassifier	0.952778	0.904762	0.049618
MLPClassifier	0.952778	0.904762	0.086684

## 6. Discussion

The proposed AI-driven system demonstrated robust performance in automated blood cell anomaly detection, achieving a precision of 0.98, recall of 0.99, and F1 score of 0.98 on our primary dataset. A comparative evaluation on the ALL-IDB benchmark yielded consistent results (YOLOv11: 0.96 ± 0.02), indicating strong potential for clinical deployment. The integrated pipeline combining YOLOv11 for segmentation, ResNet50 for classification, and zero-shot learning (ZSL) for novel subclass detection represents a significant advancement over prior works, such as Hegde et al. [24] (90% precision) and Kutlu et al. [25] (94.3% precision), both in terms of accuracy and functional scope.

To address interpretability challenges in medical AI, we incorporated explainable techniques including Grad-CAM visualizations and SHAP value analysis, aligning with recent advances in XAI for healthcare [33]. The Grad-CAM heatmaps consistently highlighted morphologically significant regions (e.g., nuclear morphology in leukocytes) as key detection features, while SHAP analysis identified critical cellular characteristics (e.g., perimeter-to-area ratio, sphericity) driving classification decisions. However, certain edge cases—particularly the differentiation between “Lymphocyte T” and “Promyelocyte-N” subclasses—revealed persistent challenges in model interpretability, as evidenced by elevated false negative rates in ZSL predictions (Section 5).

While our curated dataset of 28,532 augmented images represents a substantial resource for hematological AI research, two limitations merit discussion. First, the absence of detailed inter-rater reliability metrics and institutional review board approvals for all data sources suggests opportunities to strengthen methodological rigor. Second, the current validation framework, while incorporating fivefold cross-validation, would benefit from the inclusion of additional external datasets beyond ALL-IDB to more comprehensively assess generalizability. The observed misclassifications in rare erythrocyte variants (e.g., “Target” cells) underscore the ongoing challenges posed by class imbalance and imaging artifacts in clinical samples.

From a computational perspective, the system’s optimized inference latency (50 ms/image on Tesla T4 hardware) demonstrates technical feasibility for point-of-care deployment. However, the absence of real-world clinical validation against manual microscopy remains a critical gap. Future comparative studies should quantify diagnostic concordance rates with hematologists across diverse healthcare settings. Similarly, while statistical validation confirmed significant improvements over baseline models (*p* < 0.01, Cohen’s d: 0.9–1.5), the clinical relevance of these effect sizes requires further investigation through controlled trials.

## 7. Conclusions and Future Work

This study presents an integrated AI framework for automated hematological analysis, combining state-of-the-art computer vision architectures (YOLOv11, ResNet50) with zero-shot learning capabilities. The system achieves high diagnostic accuracy (F1 score: 0.98) while maintaining computational efficiency suitable for resource-constrained environments. Key innovations include the following:

- A unified pipeline addressing segmentation, classification, and novel anomaly detection.
- Implementation of explainable AI techniques for clinical interpretability.
- Optimization for rapid inference (50 ms/image) without sacrificing accuracy.

Three primary limitations guide our future research directions:

1. **Clinical validation**: Pending trials comparing system performance against board-certified hematologists across diverse healthcare settings.
2. **Technical enhancements**: Integration of focal loss approaches [29] to address class imbalance and lightweight architectures like EfficientNet [17] for edge deployment.
3. **Data diversity**: Expansion of training corpora to include (a) broader demographic representation and (b) standardized ethical documentation.

The system’s current capabilities, while promising, represent an initial step toward AI-augmented hematology. Subsequent development will focus on three key areas: (1) implementation of natural language interfaces for automated report generation, (2) multi-center validation studies, and (3) integration with emerging telehealth platforms. These advances will be guided by the principle that diagnostic AI should enhance, rather than replace, clinician expertise—particularly in underserved regions where our technology may have the greatest impact.

## Figures and Tables

**Table 1:** Overview of white blood cells (WBCs) and their characteristics.

Category	Details
Role in Immunity	WBCs are the primary effectors of immunity, acting as protective cells against various forms of aggression.
Alternative Name	White blood cells are also known as leukocytes.
Classification	WBCs are classified into the following cells: Polymorphonuclear (PMNs) cells; Monocytes and lymphocytes.
Origin	All blood cells originate from a single multipotent cell in the bone marrow.
Development Process	Maturing cells acquire structural and functional properties during hematopoiesis.

**Table 2:** Comparison of related works.

Study	Approach	Advantages	Limitations/Interpretability
Hegde et al. (2019) [24]	Transfer learning for WBC classification	90% accuracy; scalable for large datasets	No real-time analysis; WBC-only focus
Kutlu et al. (2020) [25]	CNN for partial WBC recognition	94.3% accuracy; handles overlapping cells	Limited to normal WBC morphologies
Akalin et al. (2022) [26]	YOLOv5-Detectron2 hybrid model	3.44–14.7% accuracy gain; real-time capable	Computationally intensive
Rahman et al. (2021) [27]	Morphological RBC analysis	Effective color/shape-based detection	Single-modality approach
Gill et al. (2023) [28]	VGG19 for malaria detection	90% accuracy; automated diagnosis	Malaria-specific application
Pasupa et al. (2023) [29]	CNN with focal loss	Handles class imbalance; high F-scores	Requires hyperparameter optimization
Khan et al. (2024) [30]	RCNN for RBC classification	99% training accuracy; handles cell overlaps	Computationally demanding
Onakpojeruo et al. (2024) [31]	Conditional DCGAN + C-DCNN	99% accuracy; privacy-preserving	Neuroimaging-specific validation
Onakpojeruo et al. (2024) [32]	Pix2Pix GAN augmentation	86% accuracy; multi-class capability	Untested for hematological analysis

**Table 3:** Primary dataset composition across partitions.

Segmentation Type	Partition	Images	Classes/Image
Multi-class	Training	8380	2–10
Multi-class	Validation	2600	2–8
Multi-class	Test	1100	1–6

**Table 4:** Comparative dataset characteristics.

Characteristic	Primary Dataset	ALL-IDB
Samples	12,080 (pre-augmentation)	368
Classes	10 (cell types)	2 (normal/leukemic)
Demographics	Multi-source	Pediatric focus
Annotation Quality	99.5% complete	100% complete
Task Type	Multi-class segmentation	Binary classification

**Table 5:** Class distribution before and after augmentation.

Cell Type	Original Samples	Augmented Samples
Basophil	4200	7590
Eosinophil	3032	6804
Erythroblast	2050	6390
Intrusion	1590	5200
Lymphocyte	3620	6980
Monocyte	5307	8352
Myelocyte	4165	7490
Neutrophil	5270	8298
Platelet	4986	7916
RBC	8706	10,380

**Table 6:** Optimization results using Voxel51 on Tesla T4.

Metric	Original Model	Optimized Model	Improvement
Latency	0.0199 sec/batch	0.0035 sec/batch	3.13×
Throughput	91.66 data/sec	286.69 data/sec	3.13×
Model Size	44.98 MB	32.37 MB	−28%
Metric Drop	-	0.0363	-

**Table 7:** Comparison of YOLOv10 and YOLOv11 training and validation metrics after 200 epochs.

Metric	YOLOv10 Train	YOLOv10 Val	YOLOv11 Train	YOLOv11 Val
Box Loss	1.0	0.9	0.8	0.7
Segmentation Loss	1.5	1.2	1.2	1.0
Classification Loss	0.5	0.6	0.4	0.5
DFL Loss	0.9	1.0	0.7	0.8
Precision (B)	0.8	0.7	0.99	0.98
Recall (B)	0.7	0.6	0.98	0.97
mAP@50 (B)	0.7	0.6	0.95	0.94
mAP@50:95 (B)	0.5	0.4	0.75	0.73
Precision (M)	0.7	0.6	0.98	0.97
Recall (M)	0.6	0.5	0.97	0.96
mAP@50 (M)	0.6	0.5	0.94	0.93
mAP@50:95 (M)	0.4	0.3	0.72	0.70

**Table 8:** YOLOv10 and YOLOv11 training hyperparameters.

Hyperparameter	Value
Learning Rate	0.001
Batch Size	16
Optimizer	Adam
Epochs	200
Momentum	0.9
Weight Decay	0.0005

**Table 9:** Hyperparameters used for training the CNN-based classification model.

Hyperparameter	Value
Learning rate	0.001
Batch size	32
Number of epochs	50
Optimizer	Adam
Loss function	Binary Cross-Entropy
Activation function	ReLU (hidden layers), Sigmoid (output)
Dropout rate	0.5
Number of convolutional layers	3
Kernel size	3 × 3
Pooling type	MaxPooling (2 × 2)
Early stopping	Enabled (patience = 5)

**Table 10:** Hyperparameters used for fine-tuning the ResNet50 model.

Hyperparameter	Value
Pre-trained weights	ImageNet
Learning rate	0.0001
Batch size	32
Epochs	200
Optimizer	Adam
Loss function	Categorical Cross-Entropy
Activation (dense layers)	ReLU
Final activation	Softmax
Dropout rate	0.5
Trainable layers	Top 50% of the model
Early stopping	Enabled (patience = 10)
Data augmentation	Enabled (rotation, flip, shift)

**Table 11:** Hyperparameters used for training the zero-shot learning model.

Hyperparameter	Value
Learning rate	0.001
Batch size	16
Epochs	100
Optimizer	Adam
Loss function	Cross-Entropy
Embedding dimension	300 (GloVe)
Dropout rate	0.3
Regularization	L2 ( λ=0.01 )
Early stopping	Enabled (patience = 5)

**Table 12:** Hyperparameters used for the geometry learning model.

Hyperparameter	Value
Learning rate	0.0005
Batch size	32
Epochs	150
Optimizer	Adam
Loss function	Mean Squared Error
Scattering layers	3
Wavelet filters	8
Dropout rate	0.4
Regularization	L2 ( λ=0.01 )
Early stopping	Enabled (patience = 8)

**Table 13:** Comparative model performance analysis.

Model	Primary Dataset	ALL-IDB	Effect Size (d)	Key Advantage	Limitation
YOLOv11 (Ours)	0.98	0.95	1.5	Real-time processing	Requires GPU acceleration
ResNet50 (Ours)	0.96	0.92	1.3	Transfer learning capability	Moderate compute requirements
Geometry Learning (Ours)	0.92	0.89	1.1	Shape feature extraction	Specialized for RBC analysis
ZSL (Ours)	0.88	0.85 ± 0.03	1.0	Novel subclass detection	Needs semantic embeddings
YOLOv10 (Ours)	0.87	0.82	0.9	Balanced speed/accuracy	Lower rare-class recall
Onakpojeruo et al. (2024a) [31]	0.99 *	-	-	Synthetic data generation	Neuroimaging focus
Onakpojeruo et al. (2024b) [32]	0.86 *	-	-	Multi-class augmentation	Untested for hematology
ResViT [44]	0.82	0.78	-	Baseline comparison	Lower accuracy

* Reported accuracies from original studies (brain tumor datasets).

**Table 14:** Performance comparison of YOLOv10 and YOLOv11 on the primary dataset.

Metric	YOLOv10	YOLOv11
Precision	0.87	0.98
Recall	0.80	0.99
F1 Score	0.75	0.98

**Table 15:** Comparative analysis of blood cell analysis models.

Model	Methodology	Precision	F1 Score	Key Advantage
YOLOv10 (Ours)	Multi-label segmentation	87%	0.75	Balanced speed/accuracy
YOLOv11 (Ours)	Advanced object detection	98%	0.98	State-of-the-art performance
Hegde et al. [24]	CNN with transfer learning	90%	-	WBC classification
Kutlu et al. [25]	Partial WBC recognition	94.3%	-	Handles overlapping cells
Akalin et al. [26]	YOLOv5-Detectron2 hybrid	91.2% *	-	Real-time capability
Khan et al. [30]	RCNN with augmentation	99% **	-	Touching cell resolution

* Maximum improvement over baseline. ** Training accuracy.

## Data Availability

The private data presented in this study are available on request from the National School of Veterinary Medicine of Sidi Thabet due to privacy restrictions.

## References

[1] Blann A., Ahmed N. (2022). Blood Science: Principles and Pathology.

[2] Padalko E., Colenbie L., Delforge A., Ectors N., Guns J., Imbert R., Jansens H., Pirnay J.-P., Rodenbach M.-P., Van Riet I. (2024). Preanalytical variables influencing the interpretation and reporting of biological tests on blood samples of living and deceased donors for human body materials. Cell Tissue Bank..

[3] Walter W., Haferlach C., Nadarajah N., Schmidts I., Kühn C., Kern W., Haferlach T. (2021). How artificial intelligence might disrupt diagnostics in hematology in the near future. Oncogene.

[4] Obstfeld A.E. (2023). Hematology and machine learning. J. Appl. Lab. Med..

[5] Anilkumar K.K., Manoj V.J., Sagi T.M. (2020). A survey on image segmentation of blood and bone marrow smear images with emphasis to automated detection of Leukemia. Biocybern. Biomed. Eng..

[6] Allgaier J., Mulansky L., Draelos R.L., Pryss R. (2023). How does the model make predictions? A systematic literature review on the explainability power of machine learning in healthcare. Artif. Intell. Med..

[7] Chhabra G. (2018). Automated hematology analyzers: Recent trends and applications. J. Lab. Physicians.

[8] Malgoyre A., Bigard X., Alonso A., Sanchez H., Kelberine F. (2012). Variabilité des compositions cellulaire et moléculaire des extraits de concentrés plaquettaires (platelet-rich plasma, PRP). J. Traumatol. Sport.

[9] Xu Y., Quan R., Xu W., Huang Y., Chen X., Liu F. (2024). Advances in Medical Image Segmentation: A Comprehensive Review of Traditional, Deep Learning and Hybrid Approaches. Bioengineering.

[10] Misra V., Mall A.K. (2024). Harnessing Image Processing for Precision Disease Diagnosis in Sugar Beet Agriculture. Crop Des..

[11] Terven J., Córdova-Esparza D.-M., Romero-González J.-A. (2023). A comprehensive review of yolo architectures in computer vision: From yolov1 to yolov8 and yolo-nas. Mach. Learn. Knowl. Extr..

[12] Grandini M., Bagli E., Visani G. (2020). Metrics for multi-class classification: An overview. arXiv.

[13] Asif S., Ti W., Ur-Rehman S., Ul-Ain Q., Amjad K., Yi Y., Si J., Awais M. (2024). Advancements and Prospects of Machine Learning in Medical Diagnostics: Unveiling the Future of Diagnostic Precision. Archives of Computational Methods in Engineering.

[14] Mukherjee S. (2022). The Annotated ResNet-50: Explaining How ResNet-50 Works and Why It Is So Popular. https://towardsdatascience.com/the-annotated-resnet-50-a6c536034758/.

[15] Simonyan K., Zisserman A. (2014). Very deep convolutional networks for large-scale image recognition. arXiv.

[16] He K., Zhang X., Ren S., Sun J. Deep residual learning for image recognition. Proceedings of the IEEE Conference on Computer Vision and Pattern Recognition.

[17] Sandler M., Howard A., Zhu M., Zhmoginov A., Chen L.C. Mobilenetv2: Inverted residuals and linear bottlenecks. Proceedings of the IEEE Conference on Computer Vision and Pattern Recognition.

[18] Cao W., Wu Y., Sun Y., Zhang H., Ren J., Gu D., Wang X. (2023). A review on multimodal zero-shot learning. Wiley Interdiscip. Rev. Data Min. Knowl. Discov..

[19] Guo J., Rao Z., Chen Z., Zhou J., Tao D. (2024). Fine-grained zero-shot learning: Advances, challenges, and prospects. arXiv.

[20] Liu Z., Zhang X., Zhu Z., Zheng S., Zhao Y., Cheng J. (2021). MFHI: Taking modality-free human identification as zero-shot learning. IEEE Trans. Circuits Syst. Video Technol..

[21] Meng Y., Zhang Y., Xie J., Duan J., Joddrell M., Madhusudhan S., Peto T., Zhao Y., Zheng Y. (2024). Multi-granularity learning of explicit geometric constraint and contrast for label-efficient medical image segmentation and differentiable clinical function assessment. Med. Image Anal..

[22] Vijayalakshmi A. (2020). Deep learning approach to detect malaria from microscopic images. Multimed. Tools Appl..

[23] Yang F., Wang X., Ma H., Li J. (2021). Transformers-sklearn: A toolkit for medical language understanding with transformer-based models. BMC Med. Inform. Decis. Mak..

[24] Hegde R.B., Prasad K., Hebbar H., Singh B.M.K. (2019). Comparison of traditional image processing and deep learning approaches for classification of white blood cells in peripheral blood smear images. Biocybern. Biomed. Eng..

[25] Kutlu H., Avci E., Özyurt F. (2020). White blood cells detection and classification based on regional convolutional neural networks. Med. Hypotheses.

[26] Akalin F., Yumuşak N. (2022). Detection and classification of white blood cells with an improved deep learning-based approach. Turk. J. Electr. Eng. Comput. Sci..

[27] Rahman S., Azam B., Khan S.U., Awais M., Ali I. (2021). Automatic identification of abnormal blood smear images using color and morphology variation of RBCS and central pallor. Comput. Med Imaging Graph..

[28] Gill K.S., Anand V., Gupta R. An Efficient VGG19 Framework for Malaria Detection in Blood Cell Images. Proceedings of the IEEE 2023 3rd Asian Conference on Innovation in Technology (ASIANCON).

[29] Pasupa K., Vatathanavaro S., Tungjitnob S. (2023). Convolutional neural networks based focal loss for class imbalance problem: A case study of canine red blood cells morphology classification. J. Ambient Intell. Humaniz. Comput..

[30] Khan R.U., Almakdi S., Alshehri M., Haq A.U., Ullah A., Kumar R. (2024). An intelligent neural network model to detect red blood cells for various blood structure classification in microscopic medical images. Heliyon.

[31] Onakpojeruo E.P., Mustapha M.T., Ozsahin D.U., Ozsahin I. (2024). A comparative analysis of the novel conditional deep convolutional neural network model, using conditional deep convolutional generative adversarial network-generated synthetic and augmented brain tumor datasets for image classification. Brain Sci..

[32] Onakpojeruo E.P., Mustapha M.T., Ozsahin D.U., Ozsahin I. (2024). Enhanced MRI-based brain tumour classification with a novel Pix2pix generative adversarial network augmentation framework. Brain Commun..

[33] Saarela M., Podgorelec V. (2024). Recent applications of Explainable AI (XAI): A systematic literature review. Appl. Sci..

[34] Bai B., Yang X., Li Y., Zhang Y., Pillar N., Ozcan A. (2023). Deep learning-enabled virtual histological staining of biological samples. Light. Sci. Appl..

[35] Udayakumar D., Doğan B.E. (2024). Dynamic Contrast-Enhanced MRI. Magnetic Resonance Imaging Clinics of North America. https://www.sciencedirect.com/topics/medicine-and-dentistry/dynamic-contrast-enhanced-mri.

[36] Kaddour W. (2020). La contribution de la lecture des frottis sanguins chez les poissons: Approche par intelligence artificielle. Ph.D. Thesis.

[37] Sliti W., Ben Abdelali S.E. (2021). Optimization of a Blood Samples Reading Model for the Detection of Hematological Cells by Artificial Intelligence “Deep Learning”.

[38] El Othmani O. (2023). Système de détection des anomalies des cellules sanguines et son utilisation en télé-médecine.

[39] Souissi A. (2023). Évaluation du modèle fondationnel (You Only Look Once). Ph.D. Thesis.

[40] Labati R.D., Piuri V., Scotti F. ALL-IDB: The acute lymphoblastic leukemia image database for image processing. Proceedings of the 2011 18th IEEE International Conference on Image Processing.

[41] Wang W., Zheng V.W., Yu H., Miao C. (2019). A survey of zero-shot learning: Settings, methods, and applications. ACM Trans. Intell. Syst. Technol. (TIST).

[42] McEwen J.D., Wallis C.G.R., Mavor-Parker A.N. (2021). Scattering networks on the sphere for scalable and rotationally equivariant spherical CNNs. arXiv.

[43] Rajak A., Shrivastava A.K., Vidushi (2020). Applying and comparing machine learning classification algorithms for predicting the results of students. J. Discret. Math. Sci. Cryptogr..

[44] Tanwar V., Sharma B., Yadav D.P., Dwivedi A.D. (2025). Enhancing Blood Cell Diagnosis Using Hybrid Residual and Dual Block Transformer Network. Bioengineering.

