# Correction: El Othmani et al. AI-driven Automated Blood Cell Anomaly Detection: Enhancing Diagnostics and Telehealth in Hematology. *J. Imaging* 2025, *11*, 157

**DOI:** 10.3390/jimaging11110373

**Published:** 2025-10-24

**Authors:** Oussama El Othmani, Amine Mosbah, Aymen Yahyaoui, Amina Bouatay, Raouf Dhaouadi

**Affiliations:** 1 Computer Science Department, Military Academy of Fondouk Jedid, Nabeul 8012, Tunisia; 2 Military Research Center, Polytechnic School of Tunisia, University of Carthage, La Marsa 2078, Tunisia; 3 Laboratory of Drug Design, Faculty of Pharmacy, University of Monastir, Monastir 5060, Tunisia; 4 STRAST Research Group, Departamento de Ingeniería Telemática (DIT-UPM), Universidad Politécnica de Madrid (UPM), 28040 Madrid, Spain; 5 Hematology Laboratory, Sahloul University Hospital of Sousse, Sousse 4000, Tunisia; 6 National School of Veterinary Medicine (ENMV), University of Manouba, Sidi Thabet 2020, Tunisia

The authors wish to make the following corrections to the published paper [1]:

**Addition of Authors**

Amine Mosbah, Aymen Yahyaoui, Amina Bouatay, Raouf Dhaouadi were not included as authors in the original publication. 

**Removal of an Author**

Sami Naouali was included as an author in the original publication without enough contribution. At all authors’ request, his name has been removed from the list of authors. The corrected Author Contributions statement appears here.

Author Contributions: Conceptualization, O.E.O., A.M. and A.Y.; Methodology, O.E.O., A.M. and A.Y.; Software, O.E.O.; Validation, A.M., A.Y., A.B. and R.D.; Formal analysis, O.E.O.; Investigation, O.E.O., A.M. and A.Y.; Resources, A.M., A.Y. and R.D.; Data curation, O.E.O., A.M. and A.Y.; Writing—original draft preparation, O.E.O. and A.Y.; Writing—review editing, O.E.O., A.M. and A.Y.; Visualization, O.E.O.; Supervision, A.M. and A.Y.; Project administration, A.M. and A.Y. All authors have read and agreed to the published version of the manuscript.

**Text Correction 1**

There was an error in the original publication. Used Dataset has been revised to detail all data sources and cite them properly. A correction has been made to Sections 5.1. and 5.1.1.

*5.1. Used Dataset*

We used both private and publicly available datasets to train and test the models, encompassing diverse blood cell types and imaging conditions. Approximately 94% of the data used in this work in our primary dataset was collected from public sources from the internet, while the remaining 6% was private data composed solely of fish blood samples and collected in [36] (142 images of animal samples that originate from 13 fish taken from a fish farm in Sousse). This private dataset was extended by public data from human and animal samples in [37–39], which made up the primary dataset. To ensure robustness, this primary dataset was supplemented with a public benchmark(ALL-IDB). Preprocessing steps were applied to enhance data quality.

5.1.1. Primary Dataset Description

The primary dataset comprises images collected and annotated in [36–39]. These images originate from four sources:

- Histology slides prepared for electron microscopy;
- Blood samples collected through clinical procedures;
- Curated images from established online medical repositories;
- Public sources on the internet.

**Text Correction 2**

There was an error in the original publication. Private dataset has been revised to primary dataset throughout the paper. A correction has been made to the related paragraphs. For example, Section 5.2.

To ensure statistical rigor and reproducibility, we implemented the following evaluation framework, addressing the reviewer’s request for a robust validation strategy:

- **Fivefold Stratified Cross-Validation**:
  – Fixed random seed (42) for reproducible splits.
  – Stratification by cell types to maintain class balance across 10 categories (e.g., basophil, eosinophil, etc.).
  – A 80:20 train/validation ratio per fold (e.g., 6704 training and 1676 validation images per fold for the primary dataset pre-augmentation; 22,826 training and 5706 validation images post-augmentation).
- **L2 Regularization** (λ=0.01):
  – Applied to both CNN and Transformer components (e.g., in YOLO and ResNet50 models).
  – Penalty strength tuned via grid search on validation folds.
  – Normalized by feature counts to ensure consistency across. models.
- **Held-out Test Set**:
  – A total of 1100 images (primary dataset) and 20% of ALL-IDB (e.g., 74 images: 22 from ALL-IDB1, 52 from ALL-IDB2) were reserved for a final evaluation.
  – Balanced across cell types (10 classes for the primary dataset, 2 classes for ALL-IDB).
  – Never used during training or hyperparameter tuning.

**Text Correction 3**

There was an error in the original publication. The words “noted in Review 2”, “Review 4”, “noted in Review 4”, should be deleted. A correction has been made to Section 6. Discussion, Paragraphs 3 and 4.

While our curated dataset of 28,532 augmented images represents a substantial resource for hematological AI research, two limitations merit discussion. First, the absence of detailed inter-rater reliability metrics and institutional review board approvals for all data sources suggests opportunities to strengthen methodological rigor. Second, the current validation framework, while incorporating fivefold cross-validation, would benefit from the inclusion of additional external datasets beyond ALL-IDB to more comprehensively assess generalizability. The observed misclassifications in rare erythrocyte variants (e.g., “Target” cells) underscore the ongoing challenges posed by class imbalance and imaging artifacts in clinical samples.

From a computational perspective, the system’s optimized inference latency (50 ms/image on Tesla T4 hardware) demonstrates technical feasibility for point-of-care deployment. However, the absence of real-world clinical validation against manual microscopy remains a critical gap. Future comparative studies should quantify diagnostic concordance rates with hematologists across diverse healthcare settings. Similarly, while statistical validation confirmed significant improvements over baseline models (*p* < 0.01, Cohen’s d: 0.9–1.5), the clinical relevance of these effect sizes requires further investigation through controlled trials.

**Missing Citation**

In the original publication, References [36–39] were not cited. The citation has now been inserted in Sections 5.1 and 5.1.1.

[36] Kaddour, W. La contribution de la lecture des frottis sanguins chez les poissons: Approche par intelligence artificielle. Ph.D. Thesis, National School of Veterinary Medicine (ENMV), University of Manouba, Sidi Thabet, Ariana, Tunisia, 2020.
[37] Sliti, W.; Ben Abdelali, S.E. *Optimization of a Blood Samples Reading Model for the Detection of Hematological Cells by Artificial Intelligence “Deep Learning”*; End of Year Project Report; Tunisian Military Academy: Fondouk Jedid, Nabeul, Tunisia, 2021.
[38] El Othmani, O. *Système de détection des anomalies des cellules sanguines et son utilisation en télé-médecine*; End of Study Project Report; Tunisian Military Academy: Fondouk Jedid, Nabeul, Tunisia, 2023.
[39] Souissi, A. Évaluation du modèle fondationnel (You Only Look Once). Ph.D. Thesis, National School of Veterinary Medicine (ENMV), University of Manouba, Sidi Thabet, Ariana, Tunisia, 2023.

**Table Legend**

In the original publication, there was a mistake in the legend for Table 3, “dataset” has been revised to “primary dataset”. The correct legend appears below.

Table 3. Primary dataset composition across partitions.

**Error in Table**

In the original publication, there was a mistake in Table 6 as published. The unit “s” has been revised to “sec”. The corrected Table 6 appears below.
**Metric****Original Model****Optimized Model****Improvement**Latency0.0199 sec/batch 0.0035 sec/batch 3.13×Throughput91.66 data/sec 286.69 data/sec 3.13×Model Size 44.98 MB 32.37 MB −28%Metric Drop -0.0363 -

**Funding**

The correct funding statement should be:

Funding: This research received no external funding.

**Data Availability Statement**

The correct Data Availability Statement should be:

Data Availability Statement: The private data presented in this study are available on request from the National School of Veterinary Medicine of Sidi Thabet due to privacy restrictions.

**Acknowledgments**

The correct Acknowledgments should be:

Acknowledgments: We sincerely thank the Military Academy of Fondouk Jedid for its precious support to the achievement of this work. We also thank the National School of Veterinary Medicine of Sidi Thabet for its invaluable support in this study. The private dataset, which accounts for approximately 6% of the total data used in this work, was primarily gathered as part of Wajih Kaddour PhD thesis [36] under the direction of Raouf Dhaouadi. We also acknowledge Amine Mosbah for supplying the blood smear slides and for his guidance as a supervisor throughout the data collection, interpretation and algorithm development phases detailed in [36].

**References**

With this correction, the order of some references has been adjusted accordingly.

The authors state that the scientific conclusions are unaffected. This correction was approved by the Academic Editor. The original publication has also been updated.
